# Relevance of Multi-Omics Studies in Cardiovascular Diseases

**DOI:** 10.3389/fcvm.2019.00091

**Published:** 2019-07-17

**Authors:** Paola Leon-Mimila, Jessica Wang, Adriana Huertas-Vazquez

**Affiliations:** Division of Cardiology, David Geffen School of Medicine, Department of Medicine, University of California, Los Angeles, Los Angeles, CA, United States

**Keywords:** multi-omics, cardiovascular disease, heart disease, systems biology, data integration

## Abstract

Cardiovascular diseases are the leading cause of death around the world. Despite the larger number of genes and loci identified, the precise mechanisms by which these genes influence risk of cardiovascular disease is not well understood. Recent advances in the development and optimization of high-throughput technologies for the generation of “omics data” have provided a deeper understanding of the processes and dynamic interactions involved in human diseases. However, the integrative analysis of “omics” data is not straightforward and represents several logistic and computational challenges. In spite of these difficulties, several studies have successfully applied integrative genomics approaches for the investigation of novel mechanisms and plasma biomarkers involved in cardiovascular diseases. In this review, we summarized recent studies aimed to understand the molecular framework of these diseases using multi-omics data from mice and humans. We discuss examples of omics studies for cardiovascular diseases focused on the integration of genomics, epigenomics, transcriptomics, and proteomics. This review also describes current gaps in the study of complex diseases using systems genetics approaches as well as potential limitations and future directions of this emerging field.

## Introduction

Coronary artery disease (CAD) is the most common cause of cardiovascular death (1). Studies conducted in twins (2, 3) and in the general population have estimated a heritability of CAD at ~40–50% (4). In addition, genome-wide association studies (GWAS) have identified more than 150 genetic loci associated with CAD risk (5–18). Although GWAS studies have been successful on identifying common DNA variation implicated in cardiovascular diseases, they provide little or no molecular evidence of gene causality. In this context, the premise that rare genetic variation could have stronger functional effects on disease manifestation still is arguable (19). This realization has motivated researchers to integrate genetics studies with additional high-throughput data designed to interrogate the transcriptome, epigenome, proteome, metabolome, etc. Recent studies have implemented the integration of multi-omics data to accelerate the identification of novel mechanisms for complex diseases and understand the dynamics of disease manifestation (20–23). The relevance of integrating multi-omics data and the current statistical tools available for data integration have been reviewed in detail elsewhere (24–34). In this review, we summarize the state-of-the-art of multi-omics studies conducted in mice and humans to understand the molecular mechanisms underlying cardiovascular diseases including CAD (35–47), stroke (42, 48), heart failure (13, 49, 50), cardiac hypertrophy (13, 51), aortic valve disease (52, 53), and heart regeneration (54). We also discuss the gaps of multi-omics studies including the utility of generating multi-omics data in animal models, the importance of sex stratification on gene discovery, the inclusion of diverse populations and the integration of metabolomics and metagenomics with other omics platforms. Finally, we discuss future directions of multi-omics approaches for cardiovascular diseases and their importance in the era of precision health.

## Multi-Omics Studies for the Investigation of Cardiovascular Disease

**Figure 1:**
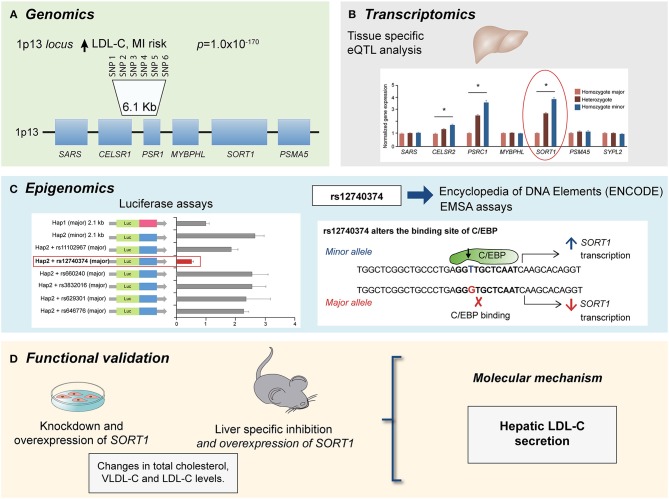
Multi-omics approach to identify the causal gene associated with LDL-C levels and CAD risk at the 1p13 *locus*. **(A)** GWAs meta-analysis showed several SNPs at the 1p13 *locus* strongly associated with LDL-C levels (*p* = 1.0 × 10^−170^) and CAD risk. The 1p13 *locus* contains several genes (squares). The most significantly associated haplotype for LDL-C comprise six SNPs in high linkage disequilibrium (LD) and is located between *CELSR1* and *PSR1* genes. **(B)** Liver eQTL analysis showed the minor haplotype significantly associated with higher expression of *CELSR1, PSR1*, and *SORT1* genes with *SORT1* gene showed the largest difference modified from Musunuru et al. (74). **(C)** By using luciferase assays and ENCODE database it was identified a common polymorphism at the 1p13 locus, rs12740374 that alters the expression of the *SORT1* gene in liver with the minor allele (T) creating a C/EBP (CCAAT/enhancer binding protein) transcription factor binding site and the major allele (G) disrupting it. The C/EBP transcriptional factor regulates the expression of hepatic genes involved in metabolism. **(D)** Functional approaches for *SORT1* using small interfering RNA (siRNA) knockdown and viral overexpression in mouse liver showed that *SORT1* results in significant changes in plasma LDL-C and very low-density lipoprotein (VLDL) particle levels by modulating hepatic VLDL secretion.

The simultaneous integration of multi-omics approaches including but not limited to genomics, epigenomics, transcriptomics, proteomics, and metabolomics (Figure 1), represents a powerful approach for understanding the mechanisms connecting identified genetic variation to cardiovascular diseases with gene causality, where many sources of variability are integrated into statistical models to identify key drivers and pathways that have the largest contribution to the disease (25). Importantly, most of the risk variants associated with CAD or other cardiovascular diseases (5, 7, 14, 17, 18, 37, 55, 56) identified by GWAS are located in noncoding regions of the genome (intronic or intergenic), suggesting that these variants are likely to affect *cis* or *trans* regulatory elements that bind transcription factors, enhancers or promoters (57). Previous multi-omic studies for CAD were mainly focused on the integration of GWAS data with global transcriptomics using eQTL analysis. In recent years, high-throughput technology have further facilitated the integration of omics data for the identification of causal genes and molecular mechanisms involved in the development of cardiovascular events in mice (13, 37, 39, 41, 58) and humans (36–39, 48) (Table 1).

**Table 1:** Studies using Multi-omics approaches for the investigation of cardiovascular diseases.

**References**	**Phenotypes**	**Population of study**	**Omic strategy**	**Tissue**	**Analysis strategy**	**Main findings**	**Genes involved**	**Functional confirmation**
Santolini et al. (13)	Isoproterenol-induced cardiac hypertrophy and heart failure	Mice (HMDP) 100 genetically diverse strains of mice	**Genomics** (genomic diversity) **Transcriptomics** (microarray platform Illumina)	H	Correlation-based method	Identification of 36 genes associated with severity of cardiac hypertrophy	*Rffl, Wdr1, Nppb, Atp6v0a1, Ankrd1, Eif4a1, Dtr (HB-EGF), Kcnip2, Pcdhgc4, Hes1, 4930504E06Rik, Akap9, 2310022B05Rik, Bclaf1, Ttc13, Nipsnap3b, Gss, Klhl23, Tspan17, Tnni2, Cab39l, Ptrf (Cavin-1), Dedd, 9430041O17Rik, Fgf16, Ehd2, Ppp1r9a, Kremen, Scara5, Zfp523, Nfatc1, Corin, Prnpip1, Lrrc1, AW549877*, and *Mkrn3*	Knockdown of *Hes1* reduces hypertrophy by 80–90% in neonatal rat ventricular myocytes
Foroughi Asl et al. (36)	CAD	CAD patients from the Stockholm Atherosclerosis Gene Expression (STAGE) study	**Genomics** [microarray platform, Affymetrix] **Transcriptomics** (microarray platform, Affymetrix)	B, AAW, MAM, LIV, SKLM, SF, VAF	Cis- and trans-gene regulation by GWAS risk loci across tissues and CAD phenotypes	Identification of 3 master regulators of CAD across 7 tissues	*FLYWCH1, PSORSIC3* and *G3BP1*	Knockdown of *FLYWCH1, PSORSIC3, G3BP1* genes affect cholesterol-ester accumulation in foam cells
Braenne et al. (37)	CAD	STAGE study Mice (HMDP)	**Genomics** (microarray platform, Illumina) **Transcriptomics** (microarray platform, Affymetrix)	LIV, SF, and M	GWAS and eQTL analysis	The majority of the GWAS loci for CAD affect gene expression (41%)	*LIPA, TOM1L2, GALNT4, SERPINH1, VAMP8, VAMP5, GGCX, PSCR1, CELSR2, SORT1, DRG2, C17orf39, MYO15A, TOM1L2, SREBF1, mir-224, hsa-miR-130a-5p, hsa-miR-4722-5p, hsa-miR-3198, hsa-miR-5197-3p, miR-378a-5p*	NA
Zhao et al. (48)	Carotid plaque, Stroke	Gene-expression profiles of 11 publically gene expression datasets of carotid plaque (*n* = 1,546). GWA studies of ischemic stroke from the International Stroke Genetics Consortium	**Genomics** (microarray platform, Illumina) **Transcriptomics** (microarray platform, Affymetrix)	H	Marker Set Enrichment Analysis (co-expression modules)	Seventeen co-expression modules were enriched for stroke. Enriched modules for stroke we associated with toll-like receptor pathway, homocysteine metabolism and phagosome formation and maturation	*F2, APOH*, and *AMBP*	NA
Lempiainen et al. (46)	CAD	GWAS studies and exome array studies for CAD.eQTL STAGE study	**Genomics** (microarray platform, Illumina) **Transcriptomics** (microarray platform, Affymetrix)	B, AAW, SKLM, SF, VAF	Construction of network modules for tissue-specific gene–protein interactions affected by genetic variance in CAD risk loci	Identification of modules with tissue-specific activity associated with CAD. Most of the modules were druggable. The top modules were implicated in extracellular matrix organization and disassembly, blood coagulation, or platelet degranulation/activation process	*LDLR, APOE, SCARB1, NOS3, CSNK2A1, HTRA1, LRP1, COL4A1, FN1, RELA, TNF, SHC1, LRP1, LYN, SYK, IGF1R, SHC1, IL6R, CXCR4, LCAT, VLDLR, PLTP, APP, SCH1, RELA, FN1, TNF, FN1, PCSK9, TRIB3, CXCR4*, and *CCR1*.	NA
Franzen et al. (38)	CAD	Patients with CAD from the STARNET studyRoad Epigenomics Consortium	**Genomics** (microarray platform, Illumina) **Transcriptomics** (mRNA sequencing, Illumina) **Epigenomics** (microarray platform, Illumina)	B, MAM, AOR, SF, VAF, SKLM, LIV	Cis- and trans-gene regulation across different tissues and CAD phenotypes	Tissue-specific gene-regulatory effects of CAD-associated SNPs identified by GWAS. Identification of 26 key drivers regulated in cis-trans by CAD SNPs	*FAM117B, LIPA, SARS, ATP5G1, GGCX, CARF, ICA1L, SH2B3, AC023271.1, RPL7P14, MAT2A, EDNRA, LINC00310, SLC22A5, NT5C2, FES, USP39, ADAMTS7, FURIN, PSMA5, ABCG5, CNNM2, SLC5A3, CACFD1, ZNF76, TCF21, PSRC1, and PDGFD*	NA
Liu et al. (59)	CAD	HCASMCs from 52 unrelated donors.	**Genomics** **Transcriptomics** **Epigenomics (ATAC-seq)**	HCASMCs	Jointly eQTL modeling and GWAS analyses	Identification of 5 genes that modulate CAD risk via HCASMCs.	*SIPA1, TCF21, SMAD3,FES,PDGFRA*	NA
Haitjema et al. (42)	CAD, Stroke	GWAS of METASTROKE and CARDIoGRAMplusC4D	**Genomics** (microarray platform, Illumina) **Transcriptomics** (mRNA sequencing, Illumina) **Chromatin Organization** (4C sequencing, Illumina)	M, CEC	Association of eQTLs with chromatin interaction	Integrative analysis of gene expression and chromatin conformation to elucidate mechanisms involved in atherosclerosis	*MIA3, PSRC1, SORT1, GGCX, VAMP5, VAMP8, NBEAL1, WDR12, MRAS, PHACTR1, TRIB1, CDKN2A, CDKN2B, KIAA1462, LIPA, COL4A1, COL4A2, PEMT, RASD1, SMG6, UBE2Z, LDLR*	NA
Lee et al. (60)			H					
Meder et al. (49)	Heart failure	135 patients with dilated cardiomyopathy 31 control subjects	**Transcriptomics** (mRNA sequencing, Illumina) **Epigenomics** (microarray platform, Illumina)	H, B	Methylation-expression quantitative trait locus analysis	Integration of methylation and gene expression data identified enrichment of cell adhesion, cardiac development, and muscle function in HF	PLXNA2, RGS3, NPPA, NPPB, B9D1, doublecortin-like kinase 2 *and* neurotrimin	NA
Rask-Andersen et al. (61)	Hypertension MI Stroke Thrombosis Arrhythmia	729 subjects from the Northern Sweden Population Health Study	**Epigenomics** Illumina Infinium 450 BeadChip	B	Integration of EWAS and ChIA-PET data	Identification of 196 genes associated with cardiac-related traits	ESRRG, ST6GALNAC5, RYR2, NMNAT2, EPHA2, TGFB2, ABCG5, FMNL2, DYSF, MEIS1, MECOM, WNT7A, SOX2, HAND2, F2RL1, KCNN2, ME1^*^	NA
Dekkers K et al. (62)	Blood lipids	3,296 subjects from the Biobank Based Integrative Omics Study	**Transcriptomics** **Epigenomics**	B	Integration of EWAS and gene expression	Identification of CpGs associated with the expression of lipids	*CPT1A and SREBF1 (TGs)* *DHCR24 (LDL-C)* *ABCG1 (HDL-C)*	NA
Howson JMM, et al. (43)	CAD	88,192 CAD cases 162,544 controls including CARDIoGRAMplusC4D database	**Genomics** (microarray platform, Illumina, Affymetrix) **Transcriptomics** (microarray platform, Illumina) **Epigenomics** (microarray platform, Illumina) **Proteomics (**multiplexed aptamer based affinity proteomics platform, SomaLogic)	30 cells/tissues including P, B, LIV, SF, VAF, H, and DT	Genomic meta-analysis, eQTL, pQTL. Enrichment analysis (Ingenuity Pathway Analysis software)	Integrative analysis showed enrichment of genes involved in biological processes active in the arterial wall as cellular adhesion, leucocyte migration, vascular smooth muscle cell differentiation, coagulation, inflammation, and atherosclerosis	*ATP1B1, NME7, CAMSAP2, DDX59, LMOD1, TNS1, TBXAS1, SERPINH1,SCARB1, TRIP4 HP, PECAM1, PROCR*	NA
Yao C, et al. (44)	CAD	6,861 subjects from the Framingham Heart Study and CARDIoGRAMplusC4D	**Genomics** (microarray platform, Illumina, Affymetrix) **Transcriptomics** (microarray platform, Affymetrix) **Proteomics (**multiplexed aptamer based affinity proteomics platform, Luminex)	P	Multi-stage strategy of proteomic analysis	pQTL analysis identified six causal proteins for CHD	*LPA, BCHE, PON1, MCAM, MPO, Cystatin C*	NA
Chen G, et al. (45)	CAD, MI	7,242 participants from the Framingham Heart Study	**Genomics** (microarray platform, Illumina, Affymetrix) **Targeted proteomics** (bead-based multiplex immunoassays, Luminex)	P	Cis- and trans-protein regulation by GWAS CAD risk loci	Identification of 210 pQTLs for 12 proteins associated with CAD and MI	*CELSR2/SORT1* locus (granulin)	NA
Fernandes, M, et al. (47)	CAD	Public databases of human samples	**Genomics** (microarray platform, Illumina, Affymetrix) **Transcriptomics** (microarray platform, Illumina) **Epigenomics** (microarray platform, Illumina) **Proteomics** (LC-MS/MS, MALDI-TOF/TOF, Thermo) **Metabolomics** (LC-MS/MS, HPLC-MS, Thermo)	ART, B, H, and LIV	Supervised development of a multi-omics integrative molecular model	Integrative analysis of omics studies showed enrichment of lipid metabolism, extracellular matrix remodeling, inflammation, and cardiac hypertrophy pathways	*LCAT, FABP1, FASN, APOA1, FASN, mir-1305 (PPARA* and *APOA1), mir-1303 (FASN)*	NA
Lau E, et al. (51)	Cardiac hypertrophy	Mice (inbred from six diverse genetic backgrounds)	**Transcriptomics** (microarray platform, Illumina) **Proteomics** (LC-MS/MS platform, Thermo) **Proteome dynamics**	H	Clustering of co-expression	Modules associated with heart hypertrophy across the mouse strains were involved in biological processes including cell adhesion, glycolytic process, actin filament organization, translation, and sodium ion transport	*ANXA2, ANXA5, COL4A2, LDHA*, and *PGAM1*	NA
Schlotter F, et al. (52)	Calcific aortic valve disease	25 human stenotic aortic valves	**Transcriptomics** (mRNA sequencing, Illumina) **Proteomics** (unlabeled and label-based tandem-mass–tagged, Thermo)	AV	Correlation of gene and protein expression differentiated between calcification stage. Protein-protein interaction	Identification of novel regulatory networks for CAVD	*SOD3. MGP, SERPINA1, VWF, C8A, C8B, SLPI, ELANE, HLA-DRA*, and *CD14*	NA
Matic LP, et al. (53)	Carotid atheroma	Patients from the Karolinska Biobank	**Transcriptomics** (microarray platform, Illumina, Affymetrix) **Proteomics** (LC-MS/MS platform, Thermo)	CP, P	Systems biology	Identification of enriched pathways for carotid atheroma including cell proliferation, nitric oxide signaling, lipoprotein, and apoptotic particle clearance, immune cell activation, chemokine secretion, blood coagulation, and extracellular matrix disassembly were dominant in plaques by transcriptomics. Extracellular matrix, heme-binding, and platelet-derived growth factor binding were the most enriched functional categories by plaque proteomics. Integrative analysis showed *BLVRB* as the only significant candidate enriched both in plaques and plasma	*BLVRB- HMOX1*	In THP-1 macrophages iron stimulated an induction of *BLVRB* and *HMOX1 was observed*.
Lalowski MM, et al. (54)	Heart regeneration	Mice	**Transcriptomics** (mRNA sequencing, Illumina) **Proteomics** (LC/MS platform, Waters) **Metabolomics** (UPLC-MS/MS platform, Metabolon)	H	Systems biology	The decrease of the heart regeneration capacity was associated with a transition from fructose-induced glycolysis under hypoxic conditions to oxidative phosphorylation, with an increase in oxidative stress, suggesting a switch from hyperplasia to hypertrophy growth. Furthermore, they found enrichment of the glycolytic pathway, mTOR, plasmalogen metabolism, methionine and histidine metabolism, lipid peroxidation, and sphingolipid signaling as novel pathways involved in heart regeneration	*Cpt I* and II, *Acaa2, Acsl1, Ecl1, Hadha, Hadhb*, and *Hsd17b10*	NA
Suhre K, et al. (35)	CAD	KORA and TwinsUK cohorts.CARDIoGRAM.	**Genomics** (microarray platform, Illumina, Affymetrix) **Metabolomics** (HPLC/MS platform, Metabolon)	B, P.	Genotype-dependent metabolic phenotypes	Some genetic *loci* that regulate blood metabolite concentrations were also associated with CAD risk (*NAT2, ABO, CPS1, NAT8, ALPL, KLKB1*). The biochemical function of the associated metabolic traits identified may support a possible role in heart disease.	*NAT2 (1-methylxanthine/ 4-acetamidobutanoate); ABO (ADpSGEGDFXAEGGGVR/ADSGEGDFXAEGGGVR); CPS1 (Glycine); NAT8 (N-acetylornithine); ALPL (ADpSGEGDFXAEGGGVR/ DSGEGDFXAEGGGVR); KLKB1 bradykinin des-arg(9)*.	NA
Feng Q, et al. (40)	CAD	59 CAD patients and 43 healthy controls	**Metabolomics** (HPLC/MS platform, Thermo) **Metagenomics** (DNA sequencing, Illumina)	P	Association of metabolites with microbiome data	Some metabolites were significantly associated with gut microbiota and CAD risk (GlcNAc-6-P, mannitol, and 15 plasma cholines). Moreover, these identified metabolites show correlations with species of intestinal microbiota (*Clostridium sp. and Streptococcus sp*.).	LPCs, glycerophosphocholines, L-Arginine, GlcNAc-6-P, and paraxanthine	NA
Cui X, et al. (50)	Chronic heart failure	53 CHF patients and 41 controls	**Metabolomics** (LC/MS platform, Thermo) **Metagenomics** (DNA sequencing, Illumina)	P	Correlation between changes in metabolites and gut microbiome associated with CHF	Enriched bacteria in CHF such as *Veillonella* were inversely correlated with cardiovascular protective metabolites such as niacin, cinnamic acid, and orotic acid. Furthermore, they found a positive correlation between the high sphingosine 1-phosphate levels and several CHF-enriched bacteria such as *Veillonella, Coprobacillus*, and *Streptococcus*.	*Veionella-* niacin, cinnamic acid, and orotic acid *Veillonella, Coprobacillus*, and *Streptococcus- sphingosine* 1-phosphate	NA
Talukdar H, et al. (39)	CAD	GWAS of CARDIoGRAMplusC4D and DIAGRAM studies. Mice (HMDP)	**Genomics** (microarray platform, Illumina, Affymetrix) **Transcriptomics** (microarray platform, Affymetrix)	AAW, SF, VAF, LIV	Marker Set Enrichment Analysis (co-expression modules). Cross-species validation using the HMDP	Identification of 30 CAD-causal regulatory gene networks interconnected in vascular and metabolic tissues	*POLR21, PQBP1, AIP, DRAP1, MRPL28, PCBD1, ZNF91*	Validation of key divers in a THP-1 foam cells
Shu L, et al. (41)	CAD T2D	GWAS data of five multi-ethnic studies including AA, EA, and HA. GWAS of CARDIoGRAMplusC4D and DIAGRAM studies. Mice (HMDP)	**Genomics** (microarray platform, Illumina, Affymetrix) **Transcriptomics** (microarray platform and mRNA sequencing, Affymetrix, Illumina) **PheWAS**	16 tissues including B, SF, ADR, ART, DT, IS, HY, LIV, LY, SKLM, TG, VE	Marker Set Enrichment Analysis (co-expression modules). Cross-species validation using cardiometabolic traits in the HMDP	Co-expression modules between CAD and T2D showed enrichment of pathways that regulate the metabolism of lipids, glucose, branched-chain amino acids, oxidation, extracellular matrix, immune response, and neuronal system. Identification of 15 key drivers associated with both CAD and T2D	*ACAT2, ACLY, CAV1, COL6A2, COX7A2, DBI, HMGCR, IDI1, IGF1, MCAM, MEST, MSMO1, PCOLCE, SPARC*, and *ZFP36*	SiRNA knockout and *in vivo* knockout of *CAV1* resulted in metabolic perturbations

*CAD, Cardiovascular Artery Disease; P, plasma; H, heart; B, blood; LIV, liver; AW, atherosclerotic arterial wall; MAM, atherosclerotic-lesion-free internal mammary artery; AOR, atherosclerotic aortic root; SF, subcutaneous fat; VAF, visceral abdominal fat; SKLM, skeletal muscle; ADR, Adrenal gland; HCASMCs, Human coronary artery smooth muscle cells; ART, Artery; DT, Digestive tract; IS, Islet; HY, Hypothalamus LY, Lymphocyte; TG, Thyiroid gland; VE, Vascular endothelium; AV, Aortic valve; M, monocytes; CEC, Coronary endothelial cells; CP, Carotid plaque*.

* *For complete list of genes see reference*.

## Success Stories of Multi-Omics Studies in Cardiovascular Diseases

Although there have been few studies integrating multi-omics profiles for the investigation of mechanisms associated with cardiovascular diseases, this approach has revealed the potential function of previously identified GWAS loci and respective mechanisms involved in these common diseases. In this section, we summarize recent studies using multi-omics approaches focusing on the integration of genomics, epigenomics, transcriptomics, and proteomics.

### Genomics, Transcriptomics, and Epigenomics

There is a large body of literature linking genetic variation with gene expression and/or epigenetic marks to understand the potential mechanisms of identified DNA variants in disease manifestation. One example on the integration of genomics with transcriptomics is a study conducted to investigate the role of the 9p21 locus (63), which was identified as one of the most significant loci for CAD in previous GWAs (64, 65). The association of CAD with this locus have been consistently replicated in multiple studies (56, 66), although the causal link of this locus remained unclear. This locus contains several genes including *CDKN2A* (encoding cyclin p14, p16), *CDKN2B* (encoding cyclin p15), *MTAP* (encoding methylthioadenosine phosphorylase), and the long non-coding RNA *ANRIL*. Integration of genetic and transcriptomic data led to the identification of *ANRIL* as the top candidate causal gene for CAD at the 9p21 region (63). Functional studies in cell lines showed possible mechanisms that could explain the role of 9p21 in CAD (67, 68). For instance, a previous study showed that alleles at the 9p21 locus were associated with different isoforms of *ANRIL* (linear or circular isoforms), where linear transcripts were associated with atherosclerosis and circular transcripts were protective against atherosclerosis. This process is mediated through the expression of multiple genes regulated in both, *cis* and *trans* (69, 70). Moreover, a recent study showed that *ANRIL* (DQ485454) is involved in endothelial cells functions important to the development of CAD including monocyte adhesion to endothelial cells, trans-endothelial monocyte migration, and endothelial cell migration (71).

Another example is the investigation of the region of the gene cluster *CELSR2-PSRC1-MYBPHL-SORT* at the 1p13.3 locus associated with low-density lipoprotein cholesterol (LDL-C) levels and cardiovascular risk (55, 72, 73). Incorporation of eQTL analysis also showed that SNPs associated with a lower risk of CAD in the 1p13.3 locus were associated with an increased gene expression of *SORT1, PSRC1*, and *CELSR2*, with *SORT1* displaying the largest expression change in the liver (73, 74). This finding allowed the construction of new hypothesis to elucidate the molecular mechanism of the 1p13.3 locus on CAD development. Studies of *SORT1* and *PSRC1* overexpression in mouse models of hyperlipidemia showed that, while *PSCR1* overexpression had no metabolic effects, *SORT1* overexpression led to a significant reduction in plasma LDL-C and very low-density lipoprotein (VLDL) particle levels by modulating hepatic VLDL secretion, suggesting an important role of *SORT1* in CAD (74). Finally, a similar omics approach was applied to identify genes associated with isoproterenol-induced hypertrophy and heart failure in the Hybrid Mouse Diversity Panel (HMDP) (13, 22, 23, 41, 75–83). The integration of genomic information and cardiac transcriptome enabled the identification of several candidate causal genes that determined the degree of cardiac hypertrophy. Specifically, *Hes1* was predicted to be involved in the progression of heart damage in cardiac hypertrophy (13). This study showed that knocking down *Hes1* in ventricular myocytes resulted in a reduction of up to 90% hypertrophy, confirming the role of *Hes1* in cardiac hypertrophy (13). More recently, several studies have demonstrated that epigenetic modifications are associated with CAD risk (38, 42, 43, 47, 49, 59, 61, 62, 84, 85), and other CVD related risk factors (61, 62, 84). Epigenetic changes that have been investigated in the context of CVD include DNA methylation (38, 43, 49), chromatin organization (42), and microRNAs (47). In recent years, efforts have been conducted to identify interactions between functional non-coding active elements of the genome and enhancers, defined as *cis*-acting DNA sequences that can increase the transcription of genes (60, 61, 86). Several methods have been developed for the identification of these interactions including, chromatin immunoprecipitation followed by high-throughput sequencing (ChIP-seq), chromatin conformation capture (3C, HiC), and most recently, chromatin interaction paired-end tagging (ChIA-PET). These technologies offer the advantage to identify genome-wide protein-DNA interactions.

### Adding Another Layer: Proteomics

The incorporation of protein expression profiles into the multi-omics studies for CAD has been less explored compared with multi-omics studies incorporating mRNA expression (43–45, 47, 51–54). This may be due to the costs and the highly specialized expertise required for instrument operation, data acquisition, and analysis of quantitative proteomics (87). Recently, Emilsson et al. showed that co-expression protein modules associated with complex diseases are highly regulated by *cis* and *trans* acting genetic variants (88). Therefore, the integration of proteomic data can add valuable information about the molecular processes involved in the development of CAD. One of the more interesting studies incorporating proteomic data in mice was conducted by Lau et al. which in addition to genomic and proteomic data, integrated protein dynamics (51). This study showed modules involved in cell adhesion, glycolytic process, actin filament organization, translation, and sodium ion transport associated with heart hypertrophy (51). In another multi-omics study conducted by Schlotter et al. for the identification of mechanisms involved in calcified aortic valve disease (CAVD) (52), the authors performed global transcriptomics and proteomics of human stenotic valves to identified novel regulatory networks in CAVD. Novel potential molecular drivers of CAVD development and progression were identified including alkaline phosphatase, apolipoprotein B, matrix metalloproteinase activation, and mitogen-activated protein kinase. Moreover, this approach also identified inflammation pathways as a significant contributor to CVD (52). This study emphasizes the relevance of extensive phenotypic characterization for multi-omics approaches to define markers associated with disease subgroups and to design more specific therapeutic strategies. In summary, these studies showed that the knowledge generated from the integration of genomics, epigenomics, transcriptomics, and proteomics could provide initial insights into the identification of mechanisms for cardiovascular diseases.

## Metabolomics and Metagenomic Studies for the Study of CAD

Metabolomics and metagenomics represent additional layers of complexity because they integrate the influences of the intake, utilization and flux of nutrients. Moreover, these omics data have proven to be useful tools for the identification of biomarkers with potential clinical applicability (89). However, studies integrating metabolomics, lipidomics, or metagenomics data in the context of CAD are limited (Table 1). In a GWAS study for metabolite levels conducted by Suhre et al. (35), the authors found several loci including *ABO, NAT2, CPS1, NAT8, ALPL, KLKB1* genes associated with both metabolites and a high risk of CAD (35). Interestingly, *KLKB1* was associated with bradykinin concentrations and with a higher CAD risk. It is known that bradykinin is a potent endothelium-dependent vasodilator that contributes to vasodilation and hypotension (90). These findings suggest that the integration of metabolomic data with other omic data can help to identify novel biomarkers for CVD diagnosis. Regarding studies integrating metagenomic data, there are only two studies for CVD so far that integrate metabolomics and metagenomics data (40, 50) (Table 1). These studies have shown species of bacteria associated with risk of CAD and plasma metabolites. For example, the bacteria *Veillonella* was associated with chronic heart failure and was also inversely correlated with known cardiovascular protective metabolites such as niacin, cinnamic acid and orotic acid (50). Nevertheless, it should be noted that these studies are only based on correlations and do not make an integrative analysis of the data, which reflects the complexity and the opportunity to develop novel statistical approaches.

## Integration of Multi-Omics, Multi-Ethnic, and Multi-Species Models of Disease

It has been suggested that comparison of “omics” data between human and animal models can provide an important contribution to the understanding of the molecular mechanism implicated in CAD (24). While studies in humans have greater translational potential, studies using animal models can help validate their biological relevance and to recapitulate the findings in humans under different environmental stimulus (22, 24, 78). This has been demonstrated in recent studies integrating multi-omics approaches for the study of CAD in both humans and animal models (39, 41). An example of a large-scale integrative multi-omic approach is the study conducted by Shu and colleagues that involved CAD and T2D GWAS data of five multi-ethnic studies (41). In this study, genetic and transcriptomic data of 16 relevant tissues for CAD were included to construct co-regulation networks for CVD and T2D (41). This network modeling allowed the identification of pathways involved in lipid metabolism, glucose, and branched-chain amino acids, along with process involved in oxidation, extracellular matrix, immune response, and neuronal system in CAD and T2D (41). Moreover, this strategy helped to dissect the molecular mechanism of *HMGCR*, identified as a top key driver for both CAD and T2D. Interestingly, the authors showed that *HMGCR* was associated with CVD and T2D in opposite directions, while genetic variants in *HMGCR* decrease CVD risk, they increase T2D risk. These findings could have important implications in the pharmacological treatment of both diseases. The integration of existing omics-data from mice and humans deposited in the cardiovascular disease database (C/VDdb), including, microRNA, genomics, proteomics and metabolomics, has recently been analyzed to identified novel drivers for CVD. In an exercise to demonstrate the utility of the C/VD database, integrative analysis of this “omics” studies showed enrichment of lipid metabolism, extracellular matrix remodeling, inflammation, and cardiac hypertrophy pathways. In addition, regulatory mechanisms mediated through miRNAs associated with the development of CAD were reported (47). Altogether, these studies illustrate that high-level integration approaches are powerful tools to extract robust biological signals across molecular layers, phenotypes, tissue types, and even species and to prioritize new therapeutic avenues for cardiometabolic diseases. Of note, there is a limited overlap in the metabolic regulators, co-expression modules and key driver gene identified across different multi-omics studies for CVD, except for markers involved in lipid metabolism which seem to be consistent among different studies. This highlights the importance of lipid metabolism in the development of cardiovascular disorders (91–93). Discrepancies of these findings could be explained by differences in the statistical tools, phenotypic characterization, ethnic origin, sex, and pathophysiological conditions (13, 23–25, 79, 94).

## Data Integration Using Freely Available Public Databases

The access to big biologic public databases allows the integration of genomic data with other “omics” including transcriptomics, proteomics and metabolomics datasets through freely available public databases such as GTEx (95) Encode (Encode project c, Roadmap (Roadmap Epigenomics Consortium, 2015), Snyderome (96) and bioRxiv, to mention a few. One of the main advantages of these databases is that allow simultaneous analysis of regulatory mechanism in different tissues, which are usually difficult to obtain in genetic studies conducted in humans. In this regard, the Genotype-Tissue Expression (GTEx) project is one of the most complete gene expression datasets currently available. This database was generated as a repository for identifying genetic variants associated with changes in gene expression (expression quantitative trait loci, eQTLs) and contains a broad tissue collection obtained from deceased donors. The last release v7, provides 11,688 transcriptomes from 714 individuals and 53 tissues. In addition GTEx also includes pathology and histology data as well as other characteristics as ethnicity, age, and sex (95). Moreover, in order to increase information about potential molecular mechanisms, the Enhancing GTEx (eGTEx) project extends the GTEx project to combine gene expression with DNase I hypersensitivity, ChIP–seq, DNA and RNA methylation, ASE, protein expression, somatic mutation, and telomere length assays (97). The Encyclopedia of DNA Elements (ENCODE) project has identified and annotated a significant amount of functional elements in the human and mice genome through diverse approaches as DNA hypersensitivity, DNA methylation, and immunoprecipitation (IP) assays of proteins that interact with DNA and RNA. The last version includes over 35 high-throughput experimental methods in > 250 different cell and tissue types, resulting in over 4,000 experiments. As GTEx database, ENCODE also includes relevant information about ethnicity, sex and age (98). Additional databases such as Roadmap (99), which has an extensive collection of DNA methylation, histone modifications, chromatin accessibility, and small RNA transcripts. The utility of these databases has been demonstrated in several studies for CAD, where their integration with genetic data facilitated the identification of regulatory mechanisms, potential targets and allows the functional validation. One example, is the prediction of the disruption of C/EBP binding site by the G allele of rs12740374 SNP using ENCODE data, functional studies showed that this variant results in a lower transcription of the *SORT1* gene in liver and a higher VLDL-secretion, explaining the association of the variant with LDL-C levels in genetic studies (Figure 1) (74). Therefore, the integration of various data frameworks could be highly successfully to understand the mechanisms implicated in disease manifestation.

## Future Directions

The identification of causal genes is a critical step toward the translation of genetic loci into biologic processes. The integration of “omic” strategies will accelerate the identification, in a more precise way, of novel molecular mechanisms implicated in CVD. This may eventually result in the characterization of novel pathways and drug targets. Although multi-omics approaches have been successfully applied for the investigation of cardiovascular diseases, the number of studies using this approach is still limited. These studies have been primarily focused on the integration of genomics, transcriptomics, epigenomics, and proteomics. Given the potential of metabolomics, metatranscriptomics, and metagenomics as tools for the identification of biomarkers with potential clinical applicability, the integration of such data will increase the understanding of cardiovascular diseases and accelerate the identification of new diagnostics or therapeutic targets (100). Finally, research efforts should be directed to the application of multi-omics and the generation of big data in more diverse populations and into the investigation of sex-specific mechanisms.

## Author Contributions

PL-M, JW, and AH-V drafted and edited the manuscript.

### Conflict of Interest Statement

The authors declare that the research was conducted in the absence of any commercial or financial relationships that could be construed as a potential conflict of interest.
